# Statistical measures of motor, sensory and cognitive performance across repeated robot-based testing

**DOI:** 10.1186/s12984-020-00713-2

**Published:** 2020-07-02

**Authors:** Leif E. R. Simmatis, Spencer Early, Kimberly D. Moore, Simone Appaqaq, Stephen H. Scott

**Affiliations:** 1 Centre for Neuroscience Studies, Queen’s University, Kingston, ON Canada; 2 Department of Biomedical and Molecular Sciences, Queen’s University, Kingston, ON Canada; 3 Department of Medicine, Queen’s University, Kingston, ON Canada

**Keywords:** Robotics, Inter-rater reliability, Intraclass correlation, Confidence interval

## Abstract

**Background:**

Traditional clinical assessments are used extensively in neurology; however, they can be coarse, which can also make them insensitive to change. Kinarm is a robotic assessment system that has been used for precise assessment of individuals with neurological impairments. However, this precision also leads to the challenge of identifying whether a given change in performance reflects a significant change in an individual’s ability or is simply natural variation. Our objective here is to derive confidence intervals and thresholds of significant change for Kinarm Standard Tests™ (KST).

**Methods:**

We assessed participants twice within 15 days on all tasks presently available in KST. We determined the 5–95% confidence intervals for each task parameter, and derived thresholds for significant change. We tested for learning effects and corrected for the false discovery rate (FDR) to identify task parameters with significant learning effects. Finally, we calculated intraclass correlation of type ICC [1, 2] (ICC-C) to quantify consistency across assessments.

**Results:**

We recruited an average of 56 participants per task. Confidence intervals for Z-Task Scores ranged between 0.61 and 1.55, and the threshold for significant change ranged between 0.87 and 2.19. We determined that 4/11 tasks displayed learning effects that were significant after FDR correction; these 4 tasks primarily tested cognition or cognitive-motor integration. ICC-C values for Z-Task Scores ranged from 0.26 to 0.76.

**Conclusions:**

The present results provide statistical bounds on individual performance for KST as well as significant changes across repeated testing. Most measures of performance had good inter-rater reliability. Tasks with a higher cognitive burden seemed to be more susceptible to learning effects, which should be taken into account when interpreting longitudinal assessments of these tasks.

## Introduction

Clinical assessment tools provide a foundation for the healthcare system, guiding patient care as well as demonstrating the benefits of novel therapeutic interventions to ameliorate the effects of disease or injury. Many advances have been made to improve clinical assessment tools, such as improved imaging techniques and novel blood-based biomarkers [1, 3]. However, assessment of brain function continues to rely largely on physical or visual inspection of the patient by a clinician. Such approaches often use coarse scales to ensure similar scores across clinicians, and also commonly have floor and ceiling effects [2, 4].

Interactive robotic systems have been used for many years for basic research to quantify upper limb sensorimotor function and provide an objective approach for quantifying neurological impairments [5–8]. These tools typically have higher sensitivity than traditional clinical instruments [9, 10]. One such tool is the Kinarm robotic platform (Kinarm, Kingston, ON, Canada) and its associated Kinarm Standard Test (KST)™ battery that quantifies upper limb sensory and motor functions, as well as cognition [11–16]. Each task generates a large number of parameters that describe spatial and temporal features of behaviour.

These robotic technologies provide considerable granularity in measuring performance, but this leads to the question of whether a change in performance reflects an actual change in an individual’s ability to perform a given task or is simply because of natural variability. For example, has performance improved significantly if an individual’s reaction time gets faster by 5% on a follow-up assessment? Additionally, does learning impact performance such that participants tend to be better when assessed a second time? The answers to these questions require knowledge of the natural variability in performance and how repeat testing impacts performance.

The objective of the present study is to quantify inter-test variability between assessments in KSTs. In the past we have collected large cohorts of healthy control participants that could be used to estimate performance variability directly, assuming that all individuals are equally capable at a given task. However, there are obvious differences in the ability of individuals to perform various sensory, motor and cognitive tasks [17–20]. Thus, our strategy is to compare performance across two repeated tests for a cohort of healthy control participants and estimate the confidence intervals of expected change based on the differences in performance between the two assessments. This approach will also allow us to determine if there are any learning effects between assessments. It will additionally provide benchmarks to use for future studies of significant change on objective robotic assessment variables. This has a wide range of potential applications, from providing a framework to quantify expected changes in novel robotic assessment tasks, to potentially assisting with quantifying the effects caused by therapeutic interventions for disease and comparing different clinical populations over time.

## Methods

### Participants

Participant recruitment was community-based (Kingston, ON, Canada), and we contacted individuals who had previously participated in Kinarm studies. Participants were excluded if they: 1) had any current, or previously diagnosed, neurological impairment, 2) they were incapable of understanding or properly completing the assessment protocol, or 3) had any upper limb impairments that negatively affected their ability to perform the required motor actions. This information was obtained from a brief interview detailing each participant’s medical history, performed to ensure eligibility. Prior to the robotic assessment, participants provided written consent. Participants in our database who had been assessed twice in a behavioural task who met the study’s inclusion criteria were also included in the cohort. This study was reviewed and approved by the Queen’s University Research Ethics Board.

### Sample size ascertainment

We performed a Monte Carlo simulation to obtain an estimated required minimum sample size of 50. Briefly, we sampled between *N* = 5 and *N* = 100 random values from a standard Normal distribution (mean = 0, standard deviation = 1) and calculated the variance of the mean and standard deviation estimated from each sample across 10,000 iterations. We observed stabilization of both the estimated mean and standard deviation at a sample size of approximately 50. The variance of the estimated value of the mean was within ±0.02 at a sample size of 50, and for comparison was within ±0.01 at a sample size of 100. The variance of the estimate of the standard deviation was within ±0.01 at a sample size of 50 and for comparison was within ±0.005 at a sample size of 100. Thus, we selected 50 as our minimum sample size to obtain a reasonably stable result in the present study that was also feasible from a data collection perspective.

### Robotic assessment

**Fig. 1:**
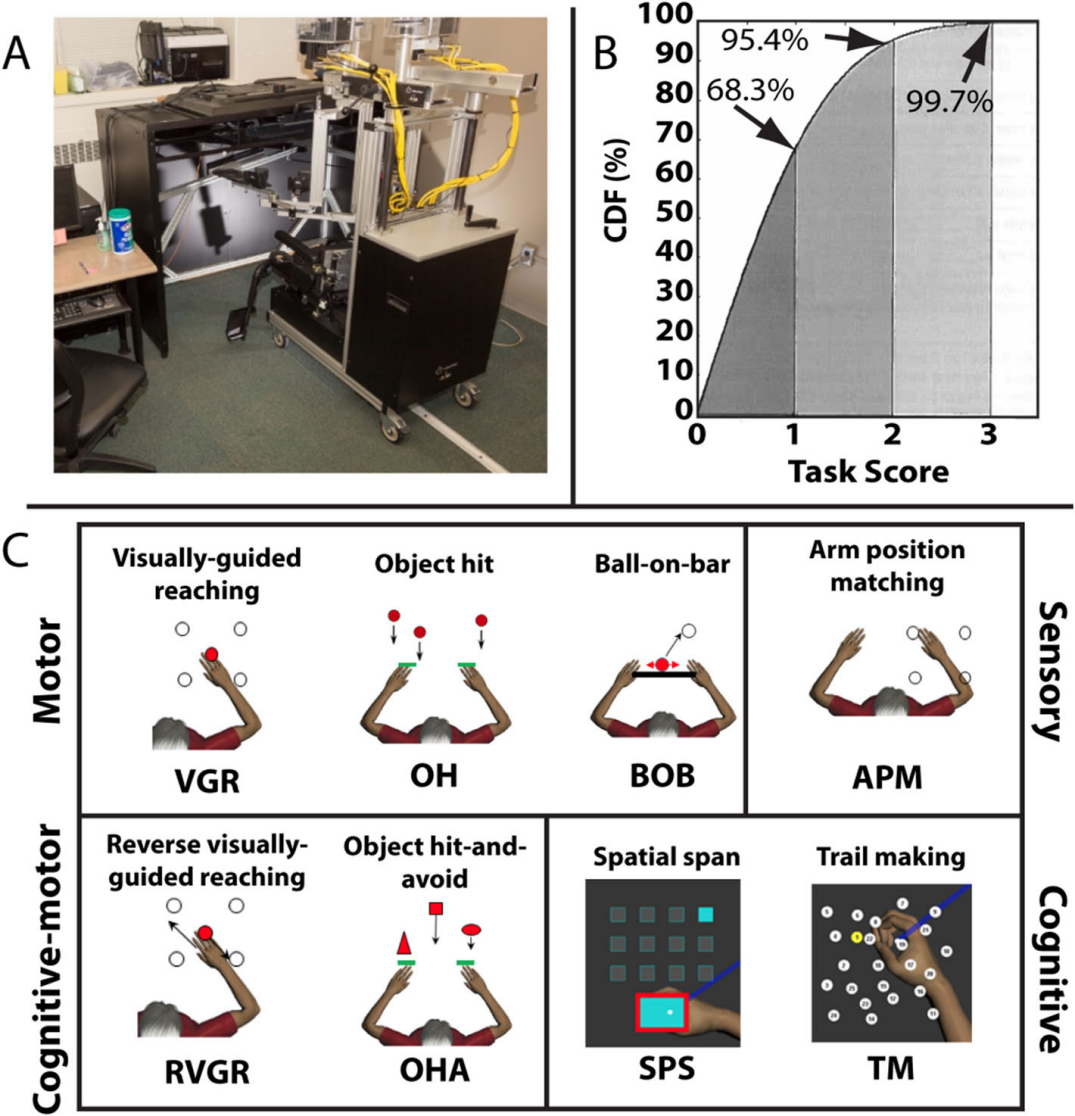
The Kinarm exoskeleton robot, tasks performed in this study, and characteristics of the Task Score distribution. **a**) Participants were seated and moved their arms in the horizontal workspace underneath a semi-transparent glass sheet. Tasks were projected onto the glass from above. Vision of the hands was obscured, however visual feedback was provided in most tasks. **b**) Participants completed 8 behavioural tasks testing motor, cognition-motor, cognitive, and sensory behavioural domains. VGR, RVGR, and APM were performed in each arm individually, yielding 2 datasets for each of these tasks. **c**) The Task Score cumulative density function (CDF) approximates that of the standard Normal distribution. A Task Score of 0 is the best, and increasing values reflect poorer performance. The distribution of Mahalanobis distance scores (M-Scores) is the same

Robotic assessment for the study was conducted on the Kinarm exoskeleton robotic platform (Kinarm, Kingston, ON, Canada). Participants were seated and their arms were placed in troughs attached to each arm that supported both the upper- and lower segments of the arms, providing full anti-gravity support. The arm troughs and seat height were adjustable to ensure that participants were comfortable and able to move their arms throughout the horizontal workspace (Fig. 1). Vision of the participant’s hands was obscured by a physical barrier; visual feedback of the hand(s) and objects was provided by a virtual reality system that was aligned with the workspace.

Participants performed each of the 8 tasks presently available in the KST battery. Detailed task descriptions are presented in Table 1. For this study, participants were evaluated on each task twice, each time by a different experienced Kinarm operator. Examinations were completed within 15 days of each other, and commonly on the same day.

**Table 1:** Task descriptions

Task	Description
**Visually guided reaching (VGR)**	VGR tests the ability to make smooth and accurate reaches. Participants were required to make quick and accurate reaches from a central target to 4 peripheral targets in sequence [11, 21].
**Object hit (OH)**	OH required participants to hit as many virtual balls away from them as possible. The task got harder as it went on, with balls falling faster. The task lasted for a fixed amount of time [12]. This task tests bimanual motor skill.
**Ball on bar (BOB)**	BOB tests bimanual coordination. Participants were required to move a ball balanced on a bar to a sequence of 4 targets, matching as many as possible in the 1 min allotted per level. In level 1 the ball was fixed to the bar but in subsequent levels the ball was able to move and fall off of the bar [14].
**Reverse visually guided reaching (RVGR)**	RVGR tests the ability to inhibit an automatic motor response. It is similar to VGR except the cursor indicating the participant’s hand position moved in the opposite direction of the hand [22] after attaining the central target.
**Object hit and avoid (OHA)**	OHA tests rapid decision-making processes. Participants had to hit two specific shapes (e.g. a vertical ellipse and a small square) and avoid 6 other distractors [13]. It is similar to OH otherwise.
**Spatial span (SPS)**	SPS tests working memory. A random sequence of square targets was displayed on a grid which participants had to recall in the same order as they were presented. Sequence length was increased 1 after a successful trial and reduced by 1 after an unsuccessful trial [23].
**Trail making (TMT)**	TMT required participants to navigate between targets labelled with numbers (1..2..etc.; variant A) or numbers and letters (1..2..etc.; variant B) in the correct sequence as quickly as possible. There were 25 targets in both variants [16, 24]. This task tests processing speed (A) and set-switching (B).
**Arm position matching (PM)**	In PM, the robot moved one of the participant’s hands and the goal was to mirror-match the position as accurately as possible using the other hand. The participant could not see where their hand was, requiring the task to be completed ‘by feel’. The task was not timed [25].

### Data normalization and task scores

Values for parameters were converted into Z-scores prior to analysis, to provide measures comparable across parameters (as opposed to varying units, e.g. seconds, metres/second). These Z-scores were additionally condensed down to Z-Task Scores and Z-M-Scores that aggregated all parameter Z-scores into convenient summaries of overall performance on a task. Z-score transforms for each task were developed from a large cohort of healthy control participants and consider the influence of age, sex, handedness, and robotic platform on performance [15, 26]. Box-Cox equations were used to normalize the distributions. Before calculation of the Z-Task Score, parameter scores were first Normalized and converted to Z-scores by an iterative process of de-skewing and outlier removal (observations |Z| > 3.29 were considered as outliers). We then used a transform [15] to convert the two-sided Z-Task Scores to “true” one-sided Task Scores. Z-Scores that had a one-sided impairment (e.g. numbers of objects hit in OH, where more was always better) were further standardized such that impairment was always considered to be a higher value. Mathematically, this was achieved by transforming the Z-scores with one-sided impairment into “Zeta-scores”. This was necessary to ensure that values with impairments in opposing directions were represented equally. For example, hitting more objects in OH (higher Z-score) is always better, whereas a lower initial movement direction angle (lower Z-score) in reaching tasks is always better. This was mathematically achieved as follows:

1 $$ Zeta=\sqrt{2}\bullet erfcinv\left(0.5\bullet \mathit{\operatorname{erfc}}\left(\frac{Z}{\sqrt{2}}\right)\right) $$

Here, *erfc* refers to the complementary error function and *erfcinv* refers to its inverse (implemented in Matlab R2018a as erfc and erfcinv functions, respectively). Equation (1) ensured that “good performance” was always represented by smaller values and “poor performance” was always represented by higher values. Z-scores with two-sided impairments were left alone (e.g. those pertaining to laterality in OH or OHA, where too much lateralization either to the left or to the right could represent impairment). Next, the root-sum square (RSS)-distance was derived:

2 $$ rssDistance=\sqrt{\sum \limits_i{Z_i}^2+\sum \limits_j{Zeta_j}^2} $$

This is effectively the Euclidean distance of all parameter Z-scores. The *rssDistance* was then converted to a Z-score using the Normalization procedures employed during parameter Z-score calculation, above. This value was referred to as the Z-Task Score. For the Z-M-Score, the distance function is not *rssDistance*, but Mahalanobis distance [27].

Finally, the one-sided Task Score was calculated:

3 $$ Task\ Score=\sqrt{2}\bullet erfcinv\left(0.5\bullet \mathit{\operatorname{erfc}}\left(\frac{ZTaskScore}{\sqrt{2}}\right)\right) $$

### Intraclass correlation

We used intraclass correlation (ICC) correlation to statistically evaluate the relationship between first and second Kinarm assessment performances. ICC conveys the degree of self-similarity of elements within the same group [28–30] and is theoretically bounded between 0 and 1 (negative values can occur in practice). For the purpose of this study, the consistency ICC metric (ICC (1, 3)) was used, which we refer to as ICC-C throughout. ICC-C is calculated as follows:

4 $$ ICC(C)=\frac{Participant\ variability}{Participant\ variability+ Measurement\ error} $$

5 $$ ICC(C)=\frac{MS_S}{MS_S+{MS}_E} $$

Where MS_S_ is the mean square (MS) between subjects and MS_E_ is the MS of remaining error. We additionally removed outliers prior to the calculation of the ICC-C, as per the following discussion in the next section. We used ICC-C as opposed to the ICC (2, 1) “absolute agreement” ICC, because ICC (2, 1) additionally accounts for systematic biases across assessments. We explicitly calculated learning effects, which are effectively systematic biases, in the present study, and so we chose not to additionally model them in the ICC calculation.

### Significant change across assessments and assessment confidence interval

Significant change thresholds (SC) and confidence intervals (CI) were estimated by first computing the difference in performance between the first and second assessments and determining the variability of these difference scores. A parameter Z-score difference (i.e. the difference between first and second assessments) exceeding ±3.2 was considered an outlier, reflecting the fact that such a large difference should only be observed 1 in 1000 data samples. These outliers were not included in any further calculations; however, we quantified the number of difference scores removed in this way. We then computed the standard deviation (SD) of the remaining difference scores, referred to as SD_diff_.

Determination of the SD_diff_ allowed the determination of both the CI and the SC. CIs were simply represented as CI = ±1.64 * SD_diff_. The choice of 1.64 as the width of the CI signifies that only 5% of healthy subjects should display such a large increase or a large decrease in performance across repeat testing. This can also be considered as approximately the 90% one-tailed confidence interval, to reflect that the most common question under consideration will be whether or not a participant had improved *or* deteriorated specifically (i.e., not the generalized question of whether someone had changed, in which case a two-tailed interval with a width of 1.96 would be more appropriate). The CI then led to the threshold for significant change (SC) in the following ways [31–35]:

6 $$ SC=\sqrt{2}\bullet CI $$

7 $$ SC=\sqrt{2}\bullet \left(\pm 1.64\right)\bullet {SD}_{diff} $$

Note that in situations in which only the pre- or post-test SD is known, and the SD of difference scores is not, the SD_diff_ may be replaced with SD_pre_*sqrt (1-ICC) = SD_post_*sqrt (1-ICC) [34, 35].

### Learning effects

Learning effects were calculated by taking the difference between first and second assessment Z-scores. We used a paired-sample t-test with α = 0.05 for the test significance level. We performed comparisons with a large number of Kinarm variables and therefore we deemed it appropriate to correct learning effect *p*-values for multiple comparisons. The relatively high number of comparisons (> 150) means that a typical Bonferroni correction for family-wise error rate will be too conservative and falsely reject some of our findings as non-significant. Therefore, we report significance after correcting for false discovery rate (FDR) using the procedure developed by Benjamini and Hochberg [36]. We indicate values that are less than 0.05 as well as those that remain significant after FDR correction.

### Simulations: CI, SC, and effect of task score transform on CI

We performed three simulations of 1) the probability that a participant is “truly impaired”, 2) that their score had “significantly changed” using the example of the Reaction Time (RT) parameter of VGR, and 3) of the effects on the CI of the conversion of the Task Score from a two-sided metric (the “Z-Task Score”) to a one-sided metric (the “Task Score”).

For 1) and 2), we fit a Gaussian curve to 7500 uniformly sampled *x* values (from − 3.75 to + 3.75, for plotting convenience) to simulate possible observations of the RT parameter, scaled either to the width of the CI or the SC. Finally, for 3), we wished to demonstrate the asymmetry induced in the CI of the one-sided Task Score by the inclusion of a CI in the two-sided Z-Task Score. Although we do not quantify these effects further in this study, and instead focus on the Z-Task Score for ease of interpretation, we believe that the consideration of the one-sided Task Score CI in the present works lays the groundwork for future studies to expand upon these ideas. We simulated *n* = 10,000 Normal random numbers with a mean of 0 and standard deviation of 1, to simulate potential Z-Task Score values. See *Data Normalization and Task Scores*, above, for further detail on Task Score calculation. We additionally incorporated the CI into the Task Score calculation:

8 $$ Task\ Score\pm CI=\sqrt{2}\bullet erfcinv\left(0.5+0.5\bullet \mathit{\operatorname{erfc}}\left(\frac{ZTaskScore\pm CI}{\sqrt{2}}\right)\right) $$

Our simulation employed a CI of ±1 for simplicity of plotting and interpretation.

### Accounting for intra-individual variability

Finally, 3 tasks (PM, RVGR, and VGR) in the current KST battery rely on participants performing multiple trials at each assessment, which are then averaged to obtain each parameter Z-score. The difference between the true unobserved mean and the mean estimated across repeated trials adds to the variability in our calculations of SC. We can estimate the influence of this intra-subject variability, and we refer to this as the intra-subject error (IS error). First, we calculated the standard error of the mean (SEM) for each assessment separately and pooled these values across all individuals. The SEM is in the same units as SD_diff_, so we calculated the final IS error by multiplying SEM by √2*1.64 so that it would be comparable to the SC (recall that SC = SD_diff_ * √2 * 1.64). Of the 3 tasks mentioned, we could extract trial-level information for RVGR, VGR, and PM. Twenty- and twenty-four, and twenty-five trials were performed for VGR, RVGR, and PM, respectively.

## Results

### Participant demographics

Demographics of all participants are summarized in Table 2. Data were collected from an average of 56 (range: 51–63) participants for each behavioural task. Fifty participants were specifically recruited for this study, whereas any additional numbers were from participants already existing in the database. All participants included in the present study completed their repeat assessments within 15 days. In total, 6 individuals had been previously assessed on a subset of the tasks presented in this work; thus, the present results for these individuals represent their second and third assessments. The intervals between previous assessments and those pertaining to the present work were [937, 482, 456, 426, 363, 233] days. We allowed their inclusions because we expected that they did not retain enough information regarding the tasks being assessed to influence their results. Additionally, a total of 10 individuals had been previously assessed in the Kinarm but on different tasks, i.e. they used the device but did not do the same tests. The intervals between these previous assessments and those of the present study were [937, 426, 233, 34, 28, 19, 13, 13, 8, 7] days. Note that these individuals completed tasks that were *not* considered in the present study. We only include reference to these individuals because they had a previous experience with the Kinarm interface.

**Table 2:** Demographics

Task	% Female	% Right-handed	Age (median [min-max])
BOB	63	85	25.0 [18–83]
OH	60	86	25.0 [18–83]
OHA	62	85	25.0 [18–83]
PM-D	65	86	24.0 [18–83]
PM-ND	65	86	24.0 [18–83]
RVGR-D	65	86	24.5 [18–83]
RVGR-ND	65	86	24.5 [18–83]
SPS	65	85	24.0 [18–83]
TMT	65	86	25.0 [18–83]
VGR-D	65	87	24.5 [18–83]
VGR-ND	65	87	24.5 [18–83]

### Significant change and confidence intervals

Table 3 displays the significant change and confidence intervals for Z-Task Score. Note that two Z-Task Score values were removed as outliers (one in each of PM-D and VGR-D). Significant change values ranged from 0.87 to 2.19, and the average significant change value was 1.51. Confidence intervals ranged from 0.61 to 1.55 for Z-Task Scores, and the average confidence interval magnitude was 1.07.

**Table 3:** Summary of data for Z-Task Scores only

Task	Outliers Removed	Significant Change	Assessment Confidence	Learning Effect	LE p-value	ICC Consistency
BOB	0	1.33	0.94	*−0.26*	0.017	0.55
OH	0	1.65	1.17	−0.18	0.15	0.49
OHA	0	1.42	1.01	*0.27*	0.018	0.64
PM-D	1	1.82	1.28	−0.01	0.95	0.29
PM-ND	0	1.72	1.21	−0.01	0.94	0.36
RVGR-D	0	1.34	0.95	−0.78^*^	< 10^−4^	0.70
RVGR-ND	0	1.79	1.27	−0.67^*^	< 10^−4^	0.67
SPS	0	1.48	1.04	−0.39^*^	0.0024	0.56
TMT	0	0.87	0.61	−0.23^*^	0.0021	0.75
VGR-D	1	1.05	0.74	−0.07	0.44	0.30
VGR-ND	0	2.19	1.55	−0.17	0.31	0.33

Learning effects are italicized if *p < 0.05* and with a * if significant after false discovery rate correction

**Fig. 2:**
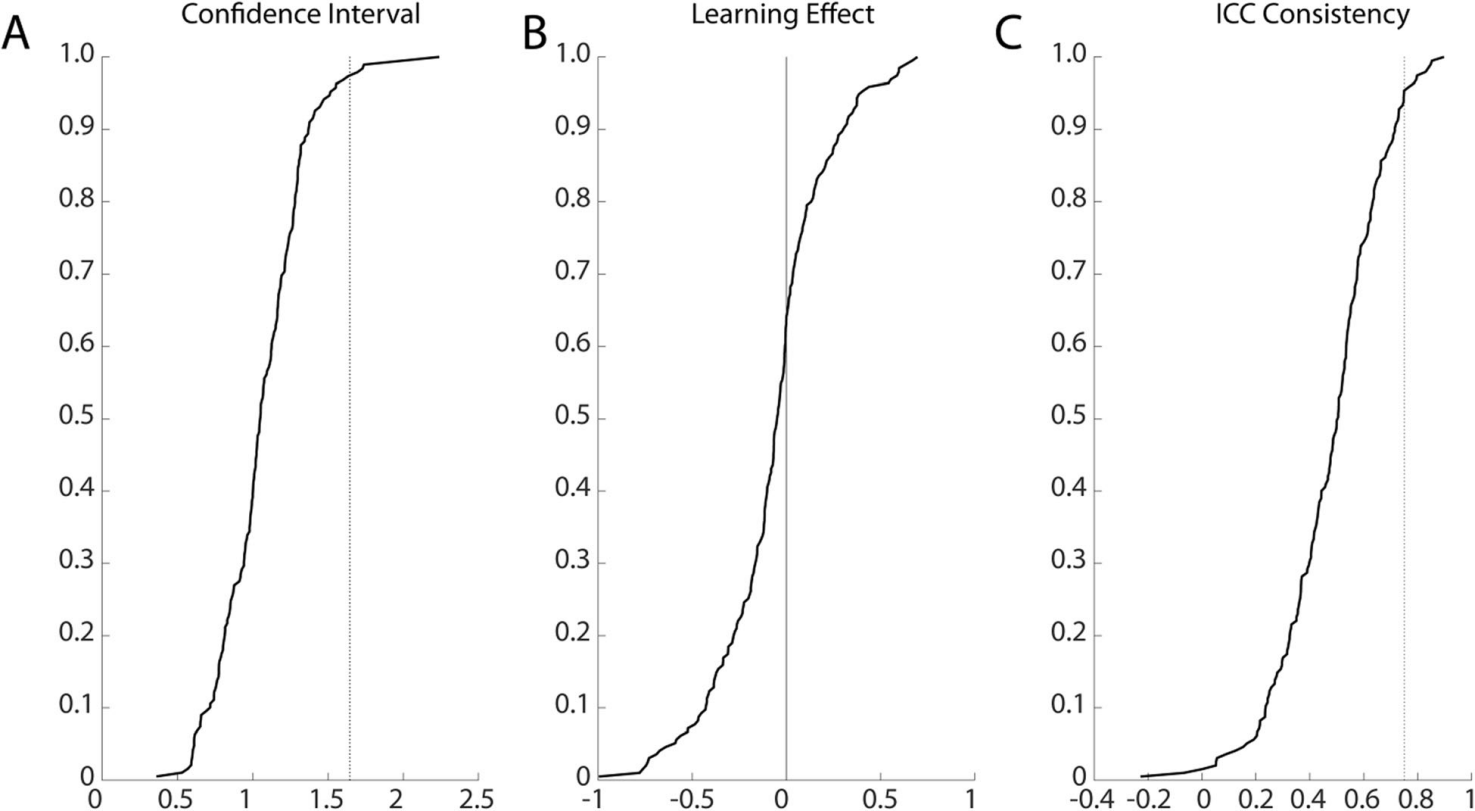
Cumulative sums of parameter metrics. **a**) Confidence intervals sorted in ascending order. Reference line is at 1.65, which is the threshold for intervals larger than expected by chance. Thus, most confidence intervals are within a reasonable range. **b**) Learning effects sorted in ascending order. Approximately 60% of learning effects were negative, indicating a lower parameter Z-score at the second assessment than the first. **c**) ICC-C values plotted in ascending order. Approximately 5% of each distribution were considered ‘good’ (> 0.75) and approximately 50% were > 0.50 (fair)

Significant change and confidence intervals for all task parameters are presented in Fig. 2a with detailed tables located in the Supplemental Material (Supplementary Tables 1–11). The mean confidence interval was 1.12 with a range from 0.60 to 2.24. Only 6 values for confidence intervals were greater than 1.64, the value if there is no difference in skill or performance between individuals. Note that significant change values are simply confidence intervals multiplied by √2, and therefore they are implicitly shifted towards higher values.

We additionally calculated IS error to understand the contribution of the variability across trials within the same assessment to the overall SC. We identified that IS values were typically on the order of 5–10% of the SC value (range of IS error to SC ratios: 0.06/1.73, i.e. 3.4%, to 0.23/1.23, i.e. 18.7%), with the VGR-ND reaction time parameters being the highest and VGR-ND path length ratio being the lowest. We report all of these values in the Supplemental file as an additional column for each of the tables for RVGR-D and RVGR-ND (Tables ST6 and ST7), and for VGR-D and VGR-ND (Tables ST10 and ST11).

### Learning effects

Learning effects ranged from 0.27 to − 0.78 for Z-Task Scores and the average learning effect was − 0.23 (Table 3). Only OHA had a positive learning effect, i.e. Z-Task Scores got slightly higher (indicating poorer performance) in this task. Six Z-Task Scores had learning effects with *p*-values < 0.05 prior to FDR correction: BOB, OHA, RVGR-D, RVGR-ND, SPS, and TMT. However, only 4 of them remained significant after correction for FDR: RVGR-D, RVGR-ND, SPS, and TMT.

The cumulative sum of the learning effects for all task parameters are presented in Fig. 2b and in the detailed tables located in the Supplement Material (Supplement Tables 1–11). The average learning effect was − 0.06 with a range from − 0.99 to 0.70. Overall, 43/167 variables met the threshold for statistical significance after correction for FDR. The task with the highest proportion of significant effects was RVGR in either arm, with 10 parameters being significant in each of the dominant and non-dominant arms, respectively. The task with the lowest number of significant learning effects was PM in either arm, with no parameters meeting the threshold for significance after FDR correction.

### ICC

We quantified ICC, using the consistency formulation (ICC (3, 1); ICC-C); see Table 3 for reference. Z-Task Score ICC-C values ranged from 0.29 to 0.75, and of these 6/11 were greater than 0.50. The task with the highest ICC-C was TMT (0.75) and the task with the lowest ICC-C was PM-D (0.29).

The cumulative sum plots of ICC-C for all parameters are presented in Fig. 2c. The parameter with the highest ICC-C values was RVGR-ND (Z-Max speed), that with the lowest ICC-C was and BOB (Z- level 3 mean bar angle). Out of all parameter ICC-C values, 12/167 (7%) were greater than 0.75 and 96/167 (57%) were greater than 0.50.

### Probabilistic interpretation of impairment and change

**Fig. 3:**
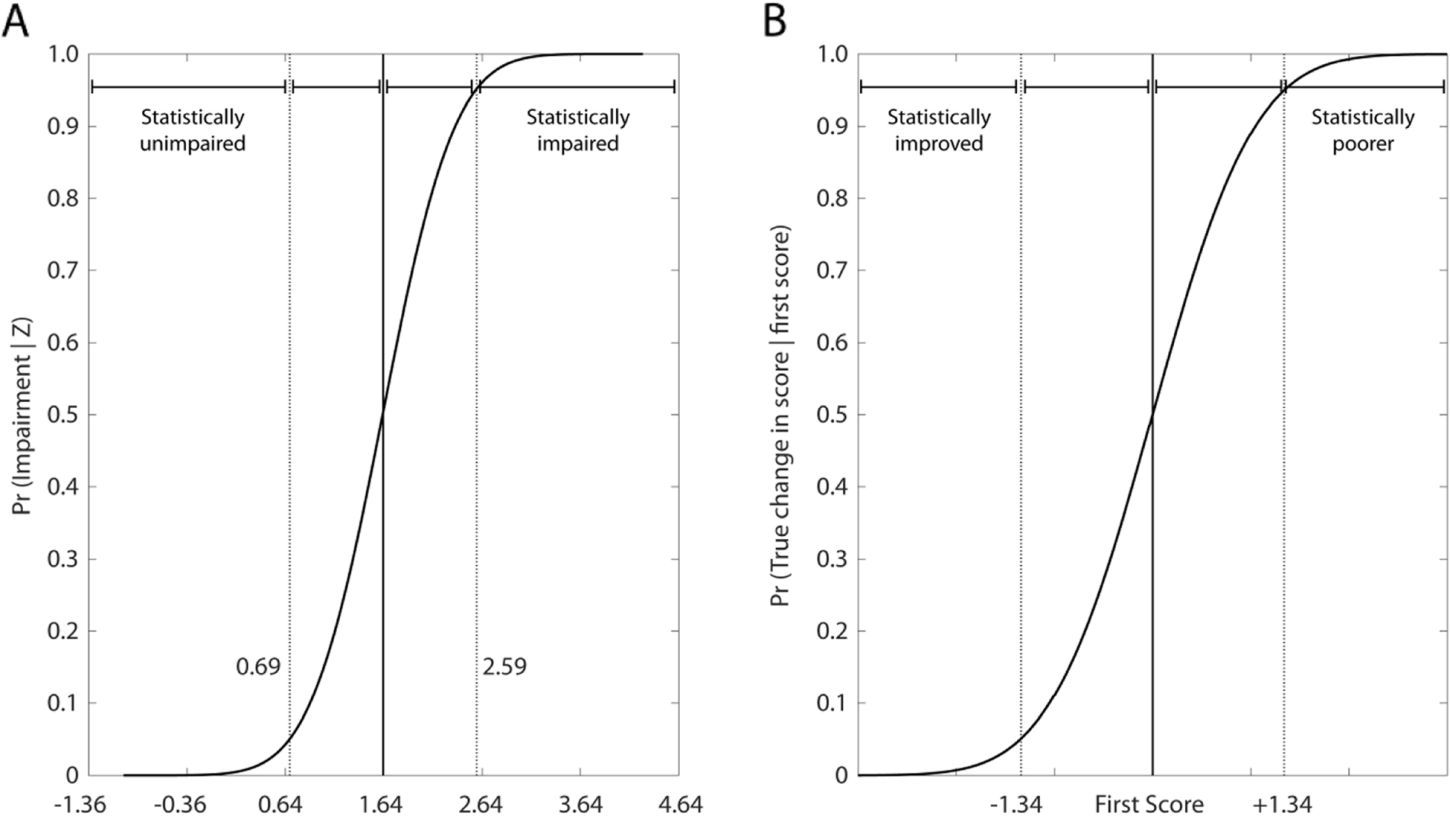
The probability of impairment given an observation, and true change given an initial score. **a**) The cumulative sum of simulated Z-reaction time scores (solid black curve), and a confidence interval (CI) of ±0.95, as was determined experimentally for this parameter. The plot is divided into 3 regions based on the likelihood that a score is *actually* impaired (i.e. is really ≥1.64) given an observed value of 1.64. A score *X* < 0.69 (1.64–0.95) is statistically unimpaired, i.e. the score is too low for there to be a reasonable probability that the true performance is impaired. A score 0.69 (1.64–0.95) ≤ *X* < 2.59 (1.64 + 0.95) is possibly impaired. A score *X* ≥ 2.59 encompasses likely impairment. **b**) The concept of **a**) can be generalized to detect a change in a follow-up assessment score *X*_*2*_ given an initial assessment score *X*_*1*_, using significant change. The plot can be divided again into 3 regions. A score *X*_*2*_ < (*X*_*1*_–1.34), i.e. the second score is less than the first score minus the significant change threshold for this parameter, is statistically improved from the first assessment. A score (*X*_*1*_–1.34) ≤ *X*_*2*_ < (*X*_*1*_ + 1.34) indicates possibly different performance at follow-up. Finally, a score *X*_*2*_ > (*X*_*1*_ + 1.34) is statistically poorer

We performed simulations of VGR Reaction Time (RT) values to depict the probabilistic interpretation of our CI and SC results in terms of identifying impairments and quantifying significant change (see Fig. 3). There is a confidence interval (CI) of performance associated with every potential score, and so it is equally probable that an individual with an RT score of 1.64 at a single assessment is actually below (not impaired) or above (impaired) the threshold of 1.64. In RT, we found that the CI was 0.95, and thus the SC was 1.34. One can identify 3 key regions of interest in Fig. 3a: 1) statistically not impaired, when the probability is less than 5% that the true score is greater than 1.64, 2) possibly impaired, when the chance of impairment is between 5 and 95%, and 3) statistically impaired, when the probability of impairment is greater than 95%. Similarly, Fig. 3b depicts the way that this same statistical approach can be used to identify whether an individual has improved/degraded between two assessments using SC criteria.

### Effects of one-sided transforms on Z-task score CI

**Fig. 4:**
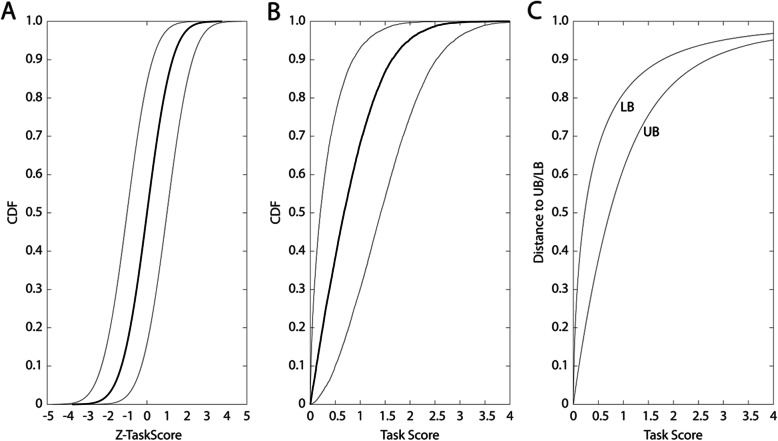
After conversion to a one-sided metric, the confidence intervals for the Task Score (and M-Score) become asymmetric. **a**) A simulated distribution of *n* = 10,000 Z-scores drawn from the standard Normal distribution (μ = 0, σ = 1). The cumulative density function (CDF) is plotted as a thick black line. Lower and upper bounds of the confidence interval (±1 for simplicity) are plotted in thin black lines. **b**) Conversion of the Z-Task Score to the true Task Score causes the CDF of **a**) to compress to the right, such that all values below zero become positive (thick black line). A Task Score of 1 has ~ 68.3% of the area of the curve underneath it, comparable to the area underneath ±1 of the standard Normal CDF. Upper and lower bounds of the confidence interval are plotted as thin black lines. The confidence interval is now asymmetric. **c**) The distance between the upper bound (UB) of the confidence interval to the Task Score grows more slowly than the distance between the lower bound (LB) of the confidence interval and the Task Score (thin black lines). The distance from the Z-Task Score to both the UB and the LB asymptotes to ±1, which corresponds to that of the original Z-Task Score in panel **a**

Finally, we considered the effects of performing a the transformation between the two-sided Z-Task Score and the one-sided Task Score that has been reported on previously [15]; see Fig. 4. Here, we calculated a CI for the Z-Task Score (and Z-M-Score, although the implications are identical given the similarity of the transformations for these two metrics). Figure 4a depicts the symmetry of a CI of ±1 about simulated Z-Task Scores with a mean of 0 and standard deviation of 1. Figure 4b depicts the effect of performing the one-sided transform from Z-Task Score to Task Score (Methods, eq. 3). The confidence intervals grow non-uniformly and are in fact a function of the Z-Task Score (and, by extension, Task Score) itself. Thus in this situation, the CI is not a fixed value. This is also demonstrated in Fig. 4c, which goes further and identifies that the upper bound (UB) and lower bound (LB) of the Task Score CI grow unequally, with the LB always growing more quickly than the UB.

## Discussion

In this work, we quantified confidence intervals, significant change, learning effects, and ICC-C (consistency type; referred to elsewhere as ICC (3, 1)) for repeated Kinarm assessments performed within 15 days of each other. Our primary objective was quantifying confidence intervals and corresponding thresholds for significant change across all Kinarm parameters. We determined that the confidence intervals averaged approximately 1.12 across Z-Task Scores and 1.07 across all parameters. These values are less than the 95% one-tailed range predicted for the entire healthy cohort (1.64).

Other prior work has investigated the reliability of various kinematic parameters post-stroke using different tools [37–39]. These studies had participants complete tasks that tested similar domains to those in the present study. For example, Rinderknecht et al. [37] employed a 2-alternative/forced-choice task to test proprioception (different from our approach but a similar underlying construct was targeted). These studies generally reported much higher ICCs than we did in the present study (on the order of 0.80 to 0.98 typically). Across these other studies, the constant factor was that individuals with stroke were assessed. A previous Kinarm study also identified high ICCs in stroke patients ranging between 0.75–0.99 [13]. We reported lower ICCs than these other studies; however, this difference is quite likely because we tested a cohort of healthy individuals. Recall that ICC models the ratio of (participant variance) / (participant variance + error variance). With this definition in mind, it is clear that if the study population is more variable relative to the amount of error, then the ICC will increase. Stroke is a heterogeneous clinical diagnosis, and so it is reasonable to expect that stroke cohorts would be more variable than a healthy cohort, leading to higher ICC values. A Kinarm study on an adult athletic population found results that were sometimes similar to those reported in our study; for example, results for the Total Hits parameter in OH were similar in terms of ICC, learning effect, and confidence interval. However, other parameters such as the Test Time parameter in TMT differed substantially [40]. The ICCs found in our current study were similar to a Kinarm study of pediatric athletes [16]. It is possible that simple differences in the study cohorts, such as age or training to do specific motor tasks as in sports, may account for differences in test-retest findings.

Knowledge of the confidence intervals of each parameter allows us to not only categorically identify if an individual’s performance falls above or below some impairment threshold, but also the probability of impairment relative to that threshold. We have commonly identified participants as impaired in the KSTs based on whether they performed worse than 95% of healthy controls [41, 42]. For example, we defined that an individual would be impaired in reaction time for VGR if they had a Z score greater than 1.64. However, as shown here, there is some variability in how a given participant performs a task. Thus, there is a confidence interval of performance associated with every potential score. This approach allows us to add a probabilistic component to the assessment and the detection of change between assessments. In some ways, the consideration of impairment as a continuum as opposed to a hard threshold is analogous to the approaches to statistical inference taken by Fisher compared to those of Pearson and Neyman [43, 44]. This probabilistic approach to detecting impairment and change may facilitate future machine learning-based approaches to detecting change and impairment, by allowing a richer range of information to be used than simple binary 1/0 values for “impaired or unimpaired”. This may be especially fruitful in clinical populations that are expected to have minimal change in the magnitude of performance on a given task, or sub-impairment deviations from normal performance.

We considered the contribution that IS error made compared to SC and found that IS error was typically relatively small compared to SC, reflecting that intra-assessment variation makes up a minority of variation compared to the variation between-assessments. We observed the highest IS errors relative to SC in VGR reaction time at ~ 20%. Overall, these findings additionally suggest that external factors – i.e. those relating to the passage of time, the specific setup of the robot, perhaps other variables like caffeine consumption or fatigue – have a larger effect the variability in performance by an individual within a single session. It is important to note that the IS error that we calculated is influenced by the number of sampled trials. Each parameter derived from multiple trials is effectively an estimate of a true, unobservable, parameter mean. Increasing the number of trials would serve to not only improve the estimate of the true value, but reduce the variability of that estimate. While attractive in theory, this would dramatically increase data collection time. It would also be unnecessary as, in practice, we were able to demonstrate that even with a relatively small number of trials [20, 26–30] the value can be estimated well enough that its associated error is small (IS error) compared by the inter-assessment change threshold (SC).

Importantly, we observed learning effects in some parameters and in some Z-Task Scores. In particular, RVGR had a preponderance of significant learning effects, with 18 parameters out of 24 (across both arms) demonstrating learning effects that were significant after correction for FDR. It could be that, in this task, there is a ‘learning curve’ that affects the first few trials [45–47]. Previous evidence suggests that there are contributions of two complementary motor learning processes. These come in the form of a fast explicit learning process that adapts to task constraints, and a slower implicit component [47–49]. RVGR, being a mirror reversal task, potentially causes the greatest retention of offline motor plan changes [45]. This means that in this task in particular, many parameters may appear to be subject to a learning effect *between assessments*, when really the observed effects are being primarily driven by learning *within the first assessment*. One way to probe this may be to quantify the extent of within-test learning and remove some number of trials after which task performance stabilizes. This approach was outside the scope of the present work, and we chose to present the results from the KSTs exactly as the tasks are available to maximize the generalizability of our results to existing tasks. Future work will address within-test learning.

Another important consideration affecting the generalization of our results concerns Task Scores and M-Scores. In this work, we report results regarding the Z-Task Score and Z-M-Score, which are the Task Score and M-Score metrics prior to being converted to one-sided values ranging from 0 to +infinity. We did this because the transformation to the one-sided scores effectively compresses the distribution of two-sided Z-values (Z-Task Score, Z-M-Score) to the right to generate the one-sided Task Scores and M-Scores. Thus, within the range of values experienced by control participants such as those we tested here (~ 95% below 1.96), the confidence interval is actually much smaller for the Task Score than for the Z-Task Score. This is an important consideration for future work; it effectively states that the better the performance of an individual is on the Task Score, the less their performance needs to change for that change to be considered significant. Some clinical measures also experience this phenomenon of score-dependent variability, although not uniformly across all assessments; examples include the Expanded Disability Status Scale and the Multiple Sclerosis Impact Scale, both used in multiple sclerosis research [50].

One of the objectives of this study was to determine if standard tests of significance would be sufficient to quantify significant change between repeated Kinarm assessments or if individual skill influenced the ability to quantify change. In the former case, each parameter Z-score could be considered as a random Normally-distributed observation pulled from a distribution of participants’ parameter Z-scores at the first assessment. Any observation sufficiently far from the mean of this distribution would represent a significant change, e,g,. a Z-score > |3.2|, representing a probability of observing a value at least as extreme as *x* given the underlying distribution of *X*, i.e. P(x|X) ≤ 0.001 [for x ∈ X ~ *N* (0,1)] by random draw. However, instead we found that all Kinarm parameter confidence intervals were well below |3.2|, indicating that there is a relationship between repeated assessment performances. This is borne out as well by the fact that almost all ICC values were not near zero.

### Limitations

Our study has some limitations to address, the first of which is that we only focused on healthy individuals in this assessment. It is possible that there will be differences in patterns of learning in individuals who, for example, have had stroke, as compared to healthy controls [51, 52]. Additionally, we only focused on one platform, the Kinarm exoskeleton. There are other Kinarm platforms available that could have different inter-test variation, which should be investigated to determine if the results obtained here generalize. A small number of participants in the study had been invited back after having done the tasks previously, which could have biased results to some extent. However, we still had more participants than we determined were necessary from our initial Monte Carlo simulations (*n* = 50). Additionally, we tested participants within 15 days; however, it is unclear how these results will generalize to time points that are spread further apart. We would like to point out that of the 6 individuals mentioned that previously complete KSTs, only one completed RVGR previously (data not shown), which is the task that had the highest learning effects across several parameters. This individual completed a prior assessment with RVGR over 900 days before and therefore we assumed that their results would not be affected because the time interval was so long. We used mostly young participants who were healthy in this study. Therefore, the generalizability of our results to older healthy individuals remains an open question. Future studies should be performed on clinical populations or control participants spanning different age ranges to identify whether or not there are substantial differences in the significant change values for clinical participants compared to healthy individuals. Finally, although we estimated that the impact of the IS error on the SC, we found that it did not substantively contribute to the overall significant change threshold. The IS error was typically < 10% of the absolute value of the SC, suggesting that the dominant source of variability is change over repeated assessments, and not change within a single session. Future work will have to consider this approach in the context of clinical disorders like multiple sclerosis or Parkinson’s disease, in which there could be potentially much greater variation due to medication doses or changes in fatigue day-to-day.

## Conclusions

The present study quantifies confidence intervals, measures of significant change, as well as reliability (ICC-C) and learning effects for the present set of behavioural tasks in KST. This framework will help with the interpretability of the performance of individual subjects by providing statistical bounds for each metric of behaviour and the significance of changes in performance across repeated testing.

## Supplementary information

**Additional file 1: Table ST1.** BOB **Table ST2.** OH **Table ST3.** OHA **Table ST4.** PM-D **Table ST5.** PM-ND **Table ST6.** RVGR-D **Table ST7.** RVGR-ND **Table ST8.** SPS **Table ST9.** TMT **Table ST10.** VGR-D **Table ST11.** VGR-ND

## Data Availability

The datasets used and/or analysed during the current study are available from the corresponding author on reasonable request.

## Abbreviations

BOB: Ball On Bar task
OH: Object Hit task
OHA: Object Hit and Avoid task
RVGR: Reverse Visually-Guided Reaching task
SPS: Spatial Span task
TMT: Trail Making Task
VGR: Visually-Guided Reaching task
ICC-C: Intra Class Correlation, Consistency type
SC: Significant change
CI: Confidence Interval
KST: Kinarm Standard Tests
CDF: Cumulative Density Function
IS error: Intra-subject error
LE: Learning effect
FDR: False discovery rate
