# Correction: Statistical measures of motor, sensory and cognitive performance across repeated robot-based testing

**DOI:** 10.1186/s12984-022-01120-5

**Published:** 2023-01-16

**Authors:** Leif E. R. Simmatis, Spencer Early, Kimberly D. Moore, Simone Appaqaq, Stephen H. Scott

**Affiliations:** 1 Centre for Neuroscience Studies, Queen’s University, Kingston, ON Canada; 2 Department of Biomedical and Molecular Sciences, Queen’s University, Kingston, ON Canada; 3 Department of Medicine, Queen’s University, Kingston, ON Canada

**Correction** **:** **Journal of NeuroEngineering and Rehabilitation (2020) 17:86** **https://doi.org/10.1186/s12984-020-00713-2**

Following publication of the original article [1], there is a few changes in the Additional file which was originally published with this article; it has now been replaced with the correct file.

Then the values under the Methods and Results has been changed. So, the Methods and Results will read as follows:

Methods: We assessed participants twice within 15 days on all tasks presently available in KST. We determined the 5–95% confidence intervals for each task parameter, and derived thresholds for significant change. We tested for learning effects and corrected for the false discovery rate (FDR) to identify task parameters with significant learning effects. Finally, we calculated intraclass correlation of type ICC (3,1) (ICC-C) to quantify consistency across assessments.

Results: We recruited an average of 56 participants per task. Confidence intervals for Z-Task Scores ranged between 0.84 and 1.41, and the threshold for significant change ranged between 1.19 and 2.00. We determined that 6/11 tasks displayed learning effects that were significant after FDR correction; these 6 tasks primarily tested cognition or cognitive-motor integration. ICC-C values for Z-Task Scores ranged from 0.29 to 0.70.

Also, a text “running Dexterit-E version 3.7 software” needs to be inserted under the section “Robotic assessment”. So, the line has been changed.

from:

Robotic assessment for the study was conducted on the Kinarm exoskeleton robotic platform (Kinarm, Kingston, ON, Canada).

to:

Robotic assessment for the study was conducted on the Kinarm exoskeleton robotic platform running Dexterit-E version 3.7 software (Kinarm, Kingston, ON, Canada).

An equation number under the section “Intraclass correlation” has been changed.

from:

For the purpose of this study, the consistency ICC metric (ICC (1, 3)) was used, which we refer to as ICC-C throughout. ICC-C is calculated as follows:

to:

For the purpose of this study, the consistency ICC metric (ICC (3, 1)) was used, which we refer to as ICC-C throughout. ICC-C is calculated as follows:

The values under the section “Significant change across assessments and assessment confidence interval” has been changed. So, the paragraph will read as follows:

Significant change thresholds (SC) and confidence intervals (CI) were estimated by first computing the difference in performance between the first and second assessments and determining the variability of these difference scores. A parameter Z-score difference (i.e. the difference between first and second assessments) exceeding ± 3.2 was considered an outlier, reflecting the fact that such a large difference should only be observed 1 in 1000 data samples. These outliers were not included in any further calculations; however, we quantified the number of difference scores removed in this way. We then computed the standard deviation (SD) of the remaining difference scores, referred to as SD_diff_.

Determination of the SD_diff_ allowed the determination of both the CI and the SC. CIs were simply represented as CI = ± 1.64 * SDdiff. The choice of 1.64 as the width of the CI signifies that only 5% of healthy subjects should display such a large increase or a large decrease in performance across repeat testing. This can also be considered as approximately the 90% one-tailed confidence interval, to reflect that the most common question under consideration will be whether or not a participant had improved or deteriorated specifically (i.e., not the generalized question of whether someone had changed, in which case a two-tailed interval with a width of 1.96 would be more appropriate). The CI then led to the threshold for significant change (SC) in the following ways [31–35]:

6 $$SC=\sqrt{2 }\cdot CI$$

7 $$SC = \left( { \pm 1.64} \right) \cdot {\text{SD}}_{{{\text{diff}}}}$$

Note that in situations in which only the pre- or posttest SD is known, and the SD of difference scores is not, the SD_diff_ may be replaced with sqrt(2) * SD_pre_ * sqrt(1 − ICC) [34, 35].

There is a text change under the section “Simulations: CI, SC, and effect of task score transform on CI”. So, the text will change.

from:

We performed three simulations of (1) the probability that a participant is “truly impaired”, (2) that their score had “significantly changed” using the example of the Reaction Time (RT) parameter of VGR, and (3) of the effects on the CI of the conversion of the Task Score from a two-sided metric (the “Z-Task Score”) to a one-sided metric (the “Task Score”).

to:

We performed three simulations of (1) the probability that a participant is “truly impaired”, (2) that their score had “significantly changed” using a simulated parameter score with a CI of 0.95, and (3) of the effects on the CI of the conversion of the Task Score from a two-sided metric (the “Z-Task Score”) to a one-sided metric (the “Task Score”).

Also, there is value change under the section “Accounting for intra-individual variability”. So, the text will change.

from:

The SEM is in the same units as SD_diff_, so we calculated the final IS error by multiplying SEM by √2 * 1.64 so that it would be comparable to the SC (recall that SC = SD_diff_ * √2 * 1.64).

to:

The SEM is in the same units as SD_diff_, so we calculated the final IS error by multiplying SEM by 1.64 so that it would be comparable to the SC (recall that SC = SD_diff_ * 1.64).

There is value and text change under the sections “Significant change and confidence intervals”, “Learning effects”, “ICC”, “Probabilistic interpretation of impairment and change”. So, the passage will read as follows:

**Significant change and confidence intervals**

Table 3 displays the significant change and confidence intervals for Z-Task Score. Note that five Z-Task Score values were removed as outliers (one is PM-D, two in RVGR-D, one in SPS, and one in TMT). Significant change values ranged from 1.19 to 2.00, and the average significant change value was 1.59. Confidence intervals ranged from 0.84 to 1.41 for Z-Task Scores, and the average confidence interval magnitude was 1.12.

**Table 3:** Summary of data for Z-Task Scores only

Task	Outliers removed	Significant change	Assessment confidence	Learning effect	LE p-value	ICC consistency
BOB	0	1.19	0.84	*−* 0.33*	0.0001	0.70
OH	0	1.72	1.22	0.01	0.97	0.47
OHA	0	1.38	0.98	0.12	0.21	0.65
PM-D	1	1.70	1.20	0.09	0.52	0.44
PM-ND	0	1.41	1.00	*−* 0.04	0.74	0.40
RVGR-D	2	1.51	1.07	*−* 0.72*	< 10^–4^	0.64
RVGR-ND	0	1.72	1.22	*−* 0.76*	< 10^–4^	0.66
SPS	1	1.46	1.03	*−* 0.43*	< 10^–4^	0.57
TMT	1	1.43	1.01	*−* 0.50*	0.0003	0.44
VGR-D	0	2.00	1.41	*−* *0.35*	0.03	0.31
VGR-ND	0	1.93	1.37	*−* 0.40*	0.01	0.29

Learning effects are italicized if p < 0.05 and with a * if significant after false discovery rate correction

Significant change and confidence intervals for all task parameters are presented in Fig. 2a with detailed tables located in the Additional file 1: Tables S1–S11. The mean confidence interval was 1.08 with a range from 0.16 to 1.54. Zero values for confidence intervals were greater than 1.64, the value if there is no difference in skill or performance between individuals. Note that significant change values are simply confidence intervals multiplied by √2, and therefore they are implicitly shifted towards higher values.

**Fig. 2:**
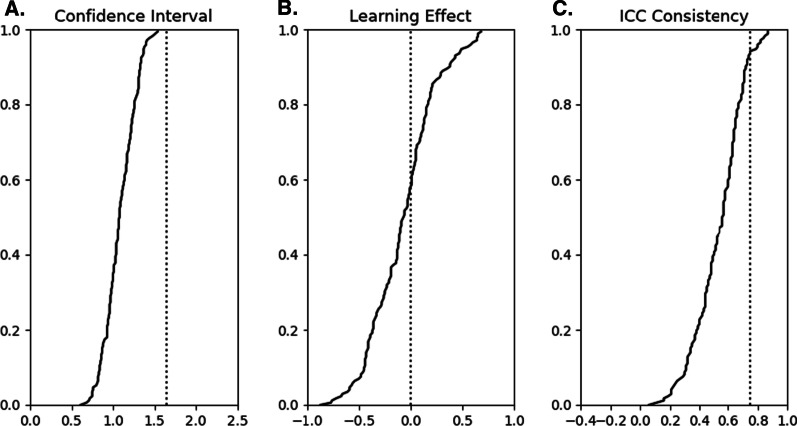
Cumulative sums of parameter metrics. **a** Confidence intervals sorted in ascending order. Reference line is at 1.65, which is the threshold for intervals larger than expected by chance. Thus, most confidence intervals are within a reasonable range. **b** Learning effects sorted in ascending order. Approximately 60% of learning effects were negative, indicating a lower parameter Z-score at the second assessment than the first. **c** ICC-C values plotted in ascending order. Approximately 5% of each distribution were considered ‘good’ (> 0.75) and approximately 50% were > 0.50 (fair)

We additionally calculated IS error to understand the contribution of the variability across trials within the same assessment to the overall SC. We identified that IS values were typically on the order of 20–30% of the SC value (range of IS error to SC ratios: 0.22/1.78, i.e., 12.4%, to 0.35/1.01, i.e., 34.7%), with the RVGR-D reaction time parameters being the highest and VGR-ND speed maxima count being the lowest. We report all of these values in the Additional file as an additional column for each of the tables for RVGR-D and RVGR-ND (Additional file 1: Table S6 and Additional file 1: Table S7), and for VGR-D and VGR-ND (Additional file 1: Table S10 and Additional file 1: Table S11).

**Learning effects**

Learning effects ranged from 0.12 to − 0.76 for Z-Task Scores and the average learning effect was − 0.30 (Table 3). OH, OHA, and PM-D had positive learning effects, i.e. Z-Task Scores got slightly higher (indicating poorer performance) in this task. Seven Z-Task Scores had learning effects with p-values < 0.05 prior to FDR correction: BOB, RVGR-D, RVGR-ND, SPS, TMT, VGR-D, and VGR-ND. However, all excluding VGR-D remained significant after FDR correction.

The cumulative sum of the learning effects for all task parameters are presented in Fig. 2b and in the detailed tables located in the Additional file 1: Tables S1–S11. The average learning effect was − 0.08 with a range from − 0.87 to 0.68. Overall, 61/172 variables met the threshold for statistical significance after correction for FDR. The task with the highest proportion of significant effects was BOB, with 10 parameters being significant. OHA, PM-D, and PM-ND each had no parameters with significant learning effects after FDR correction.

**ICC**

We quantified ICC, using the consistency formulation (ICC (3, 1); ICC-C); see Table 3 for reference. Z-Task Score ICC-C values ranged from 0.29 to 0.70, and of these 5/11 were greater than 0.50. The task with the highest ICC-C was BOB (0.70) and the task with the lowest ICC-C was VGR-ND (0.29).

The cumulative sum plots of ICC-C for all parameters are presented in Fig. 2c. The parameter with the highest ICC-C values was RVGR-D (Z-Reaction time), that with the lowest ICC-C was and TMT-B (2nd half/1st half time). Out of all parameter ICC-C values, 9/172 (5%) were greater than 0.75 and 101/172 (59%) were greater than 0.50.

**Probabilistic interpretation of impairment and change**

We performed simulations of parameter values to depict the probabilistic interpretation of our CI and SC results in terms of identifying impairments and quantifying significant change (see Fig. 3). There is a confidence interval (CI) of performance associated with every potential score, and so it is equally probable that an individual with a score of 1.64 at a single assessment is actually below (not impaired) or above (impaired) the threshold of 1.64. The CI was 0.95, and thus the SC was 1.34. One can identify 3 key regions of interest in Fig. 3a: (1) statistically not impaired, when the probability is less than 5% that the true score is greater than 1.64, (2) possibly impaired, when the chance of impairment is between 5 and 95%, and (3) statistically impaired, when the probability of impairment is greater than 95%. Similarly, Fig. 3b depicts the way that this same statistical approach can be used to identify whether an individual has improved/degraded between two assessments using SC criteria.

**Fig. 3:**
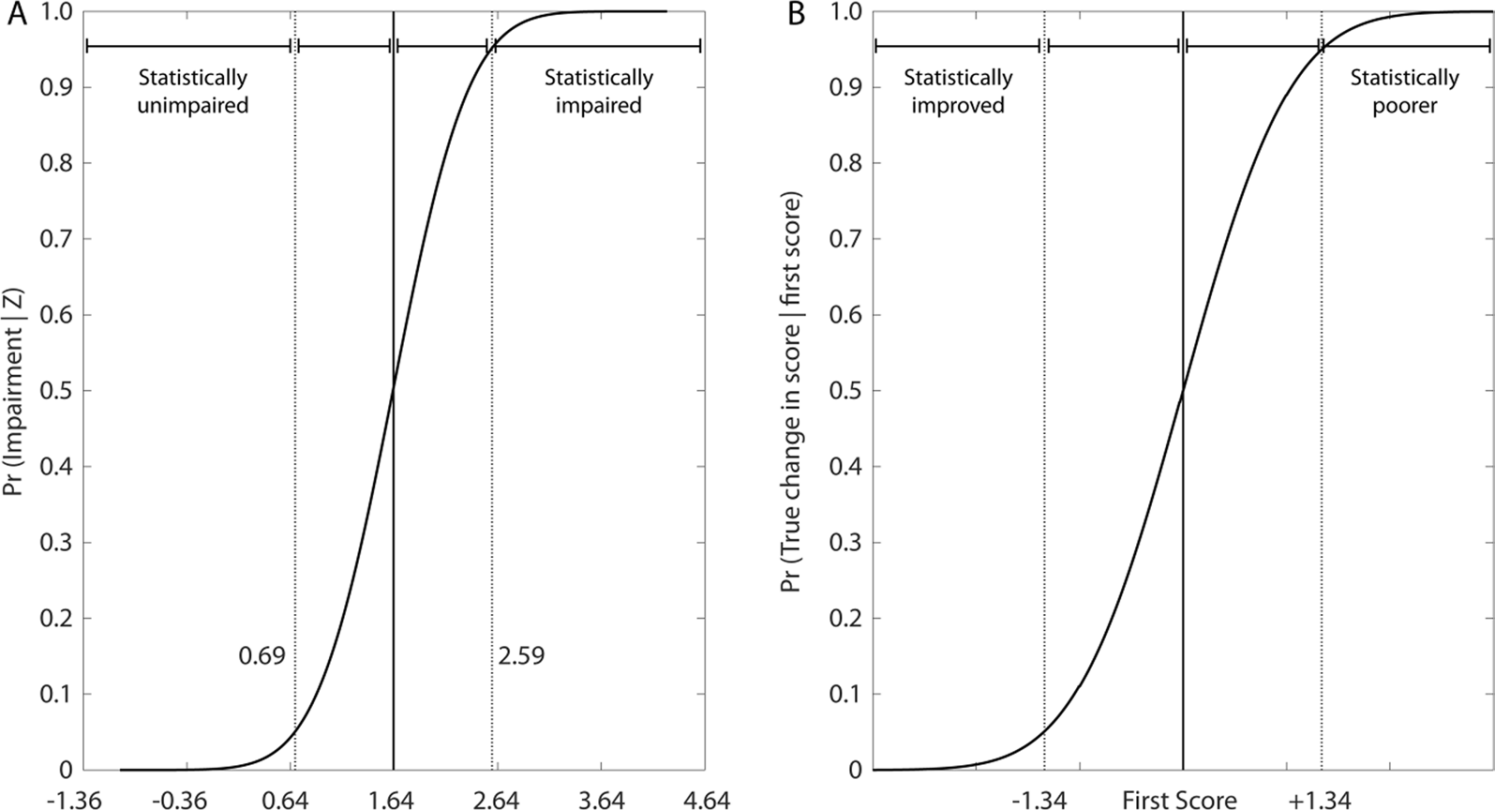
The probability of impairment given an observation, and true change given an initial score. **a** The cumulative sum of simulated scores (solid black curve), and a confidence interval (CI) of ± 0.95, as simulated for this parameter. The plot is divided into 3 regions based on the likelihood that a score is actually impaired (i.e. is really ≥ 1.64) given an observed value of 1.64. A score X < 0.69 (1.64–0.95) is statistically unimpaired, i.e. the score is too low for there to be a reasonable probability that the true performance is impaired. A score 0.69 (1.64–0.95) ≤ X < 2.59 (1.64 + 0.95) is possibly impaired. A score X ≥ 2.59 encompasses likely impairment. b) The concept of a) can be generalized to detect a change in a follow-up assessment score X2 given an initial assessment score X1, using significant change. The plot can be divided again into 3 regions. A score X2 < (X1 − 1.34), i.e. the second score is less than the first score minus the significant change threshold for this parameter, is statistically improved from the first assessment. A score (X1–1.34) ≤ X2 < (X1 + 1.34) indicates possibly different performance at followup. Finally, a score X2 > (X1 + 1.34) is statistically poorer

There is also, a value change under the section “Discussion”. So, the value in the text will change.

from:

We determined that the confidence intervals averaged approximately 1.12 across Z-Task Scores and 1.07 across all parameters.

to:

We determined that the confidence intervals averaged approximately 1.08 across Z-Task Scores and 1.12 across all parameters.

Also, there is a text change under the section “Discussion”. So, the text will change.

from:

We observed the highest IS errors relative to SC in VGR reaction time at ~ 20%.

to:

We observed the highest IS errors relative to SC in RVGR reaction time at ~ 35%.

Also, there is a the text change under the section “Discussion” and the text will change.

from:

Importantly, we observed learning effects in some parameters and in some Z-Task Scores. In particular, RVGR had a preponderance of significant learning effects, with 18 parameters out of 24 (across both arms) demonstrating learning effects that were significant after correction for FDR.

to:

Importantly, we observed learning effects in some parameters and in some Z-Task Scores. In particular, RVGR had a preponderance of significant learning effects, with 22 parameters out of 28 (across both arms) demonstrating learning effects that were significant after correction for FDR.

Also, there is a percentage change under the section “Limitation”, so the text needs to change.

from:

The IS error was typically < 10% of the absolute value of the SC, suggesting that the dominant source of variability is change over repeated assessments, and not change within a single session.

to:

The IS error was typically < 30% of the absolute value of the SC, suggesting that the dominant source of variability is change over repeated assessments, and not change within a single session.

The values in Table 3 has been changed. So, the table will read as follows:

There is a change in the x-axis for the Fig. 2. So, the figure will read as follows:

There is a small change in the label for Fig. 3. So, the label and the Fig. 3 will read as follows:

The original article has been corrected.
