# Supplementary material for: Statistical measures of motor, sensory and cognitive performance across repeated robot-based testing
Source: J Neuroeng Rehabil. 2020 Jul 2;17:86. doi: 10.1186/s12984-020-00713-2 (PMC7331240; doi:10.1186/s12984-020-00713-2)
Supplement: Supplementary file 1 — Additional file 1: Table ST1. BOB Table ST2. OH Table ST3. OHA Table ST4. PM-D Table ST5. PM-ND Table ST6. RVGR-D Table ST7. RVGR-ND Table ST8. SPS Table ST9. TMT Table ST10. VGR-D Table ST11. VGR-ND [file 12984_2020_713_MOESM1_ESM.docx]

Table ST1: BOB

| Row | Outliers  Removed | Significant Change | Assessment Confidence | Learning Effect | LE p-value | ICC-C |
| --- | --- | --- | --- | --- | --- | --- |
| Z_TaskScore | 0 | 1.33 | 0.94 | -0.26 | *0.02* | 0.55 |
| Z_1_BarLengthVariability | 0 | 1.45 | 1.03 | -0.01 | 0.90 | 0.59 |
| Z_1_HandPathBias | 0 | 1.65 | 1.16 | 0.25 | 0.10 | 0.41 |
| Z_1_HandSpeedDiff | 2 | 1.54 | 1.09 | 0.03 | 0.80 | 0.53 |
| Z_1_HandSpeedPeakBias | 0 | 1.63 | 1.15 | -0.42 | *0.01** | 0.41 |
| Z_1_LeftHandSpeedMaxima | 3 | 1.48 | 1.05 | 0.21 | 0.08 | 0.59 |
| Z_1_MeanBallSpeed | 0 | 1.59 | 1.13 | 0.56 | *<0.01** | 0.54 |
| Z_1_MeanBarAngle | 1 | 1.39 | 0.98 | -0.07 | 0.55 | 0.85 |
| Z_1_MeanLeftHandSpeed | 0 | 1.65 | 1.17 | 0.54 | *<0.01** | 0.55 |
| Z_1_MeanMovementTime | 1 | 1.46 | 1.03 | -0.64 | *<0.01** | 0.56 |
| Z_1_MeanRightHandSpeed | 1 | 1.61 | 1.14 | 0.60 | *<0.01** | 0.51 |
| Z_1_NormAbsHandSpeedDiff | 1 | 1.41 | 1.00 | -0.31 | *<0.01** | 0.51 |
| Z_1_ReactionTime_AbsDiff | 0 | 1.58 | 1.12 | -0.06 | 0.70 | 0.27 |
| Z_1_RightHandSpeedMaxima | 1 | 1.40 | 0.99 | -0.02 | 0.87 | 0.58 |
| Z_1_StdevBarAngle | 0 | 1.71 | 1.21 | -0.12 | 0.38 | 0.43 |
| Z_1_TargetsCompleted | 0 | 1.35 | 0.95 | 0.67 | *<0.01** | 0.58 |
| Z_2_BarLengthVariability | 1 | 1.48 | 1.05 | -0.12 | 0.31 | 0.68 |
| Z_2_HandPathBias | 0 | 1.52 | 1.08 | 0.23 | 0.06 | 0.34 |
| Z_2_HandSpeedDiff | 0 | 1.43 | 1.01 | 0.10 | 0.40 | 0.52 |
| Z_2_HandSpeedPeakBias | 0 | 1.42 | 1.00 | -0.10 | 0.40 | 0.64 |
| Z_2_LeftHandSpeedMaxima | 0 | 1.05 | 0.74 | -0.07 | 0.39 | 0.75 |
| Z_2_MeanBallSpeed | 0 | 1.39 | 0.98 | 0.32 | *<0.01** | 0.64 |
| Z_2_MeanBarAngle | 0 | 1.65 | 1.16 | -0.17 | 0.23 | 0.39 |
| Z_2_MeanLeftHandSpeed | 1 | 1.39 | 0.99 | 0.36 | *<0.01** | 0.63 |
| Z_2_MeanMovementTime | 0 | 1.45 | 1.03 | -0.58 | *<0.01** | 0.60 |
| Z_2_MeanRightHandSpeed | 3 | 1.31 | 0.93 | 0.37 | *<0.01** | 0.62 |
| Z_2_NormAbsHandSpeedDiff | 0 | 1.49 | 1.05 | -0.19 | 0.12 | 0.51 |
| Z_2_RightHandSpeedMaxima | 0 | 1.17 | 0.83 | -0.17 | 0.07 | 0.64 |
| Z_2_StdevBarAngle | 0 | 1.55 | 1.10 | 0.08 | 0.73 | 0.38 |
| Z_3_BarLengthVariability | 2 | 1.80 | 1.27 | -0.06 | 0.66 | 0.54 |
| Z_3_Drops_Targets | 0 | 1.59 | 1.13 | -0.49 | *<0.01** | 0.49 |
| Z_3_HandPathBias | 0 | 1.83 | 1.30 | 0.07 | 0.64 | 0.61 |
| Z_3_HandSpeedDiff | 0 | 1.14 | 0.81 | -0.16 | 0.14 | 0.56 |
| Z_3_HandSpeedPeakBias | 1 | 1.66 | 1.17 | 0.04 | 0.79 | 0.42 |
| Z_3_LeftHandSpeedMaxima | 0 | 1.14 | 0.80 | 0.09 | 0.31 | 0.71 |
| Z_3_MeanBallSpeed | 0 | 1.12 | 0.79 | -0.29 | *<0.01** | 0.80 |
| Z_3_MeanBarAngle | 0 | 1.40 | 0.99 | -0.07 | 0.69 | -0.23 |
| Z_3_MeanLeftHandSpeed | 3 | 1.14 | 0.80 | -0.08 | 0.37 | 0.80 |
| Z_3_MeanMovementTime | 0 | 1.29 | 0.91 | -0.01 | 0.92 | 0.74 |
| Z_3_MeanRightHandSpeed | 0 | 1.16 | 0.82 | -0.04 | 0.63 | 0.78 |
| Z_3_NormAbsHandSpeedDiff | 0 | 1.30 | 0.92 | -0.09 | 0.40 | 0.64 |
| Z_3_RightHandSpeedMaxima | 0 | 1.30 | 0.92 | 0.11 | 0.30 | 0.66 |
| Z_3_StdevBarAngle | 0 | 1.49 | 1.05 | -0.18 | 0.17 | 0.43 |
| Z_3_TargetsCompleted | 1 | 1.15 | 0.81 | 0.41 | *<0.01** | 0.58 |
| Z_M_score | 0 | 1.69 | 1.19 | 0.28 | *0.04* | 0.39 |

LE (learning effect) p-values are italicized if <0.05 and have an asterisk if significant after false discovery rate (FDR) correction. ICC-C = intraclass correlation coefficient, consistency type.

Table ST2: OH

| Row | Outliers Removed | Significant Change | Assessment Confidence | Learning Effect | LE p-value | ICC-C |
| --- | --- | --- | --- | --- | --- | --- |
| Z_TaskScore | 0 | 1.65 | 1.17 | -0.18 | 0.15 | 0.49 |
| Z_HandBiasOfHits | 0 | 1.49 | 1.05 | -0.07 | 0.55 | 0.48 |
| Z_HandSelectionOverlap | 0 | 1.85 | 1.31 | -0.42 | *<0.01** | 0.27 |
| Z_HandSpeedBias | 0 | 1.50 | 1.06 | -0.01 | 0.94 | 0.52 |
| Z_HandSpeedLeft | 0 | 1.10 | 0.78 | -0.12 | 0.17 | 0.67 |
| Z_HandSpeedRight | 0 | 1.10 | 0.78 | -0.11 | 0.21 | 0.72 |
| Z_HandTransition | 0 | 1.86 | 1.32 | 0.16 | 0.26 | 0.47 |
| Z_HitsWithLeft | 0 | 1.11 | 0.79 | 0.32 | *<0.01** | 0.74 |
| Z_HitsWithRight | 0 | 1.10 | 0.78 | 0.27 | *<0.01** | 0.73 |
| Z_M_score | 0 | 2.26 | 1.60 | -0.03 | 0.88 | 0.04 |
| Z_MedianError | 4 | 1.65 | 1.16 | 0.60 | *<0.01** | 0.35 |
| Z_MissBias | 1 | 1.58 | 1.11 | -0.06 | 0.63 | 0.32 |
| Z_MovementAreaBias | 1 | 2.15 | 1.52 | 0.05 | 0.77 | 0.15 |
| Z_MovementAreaLeftHand | 1 | 1.46 | 1.03 | -0.26 | *0.03* | 0.46 |
| Z_MovementAreaRightHand | 0 | 1.46 | 1.03 | -0.22 | *0.05* | 0.54 |
| Z_TotalHits | 0 | 0.85 | 0.60 | 0.38 | *<0.01** | 0.86 |

LE (learning effect) p-values are italicized if <0.05 and have an asterisk if significant after false discovery rate (FDR) correction. ICC-C = intraclass correlation coefficient, consistency type.

Table ST3: OHA

| Row | Outliers Removed | Significant Change | Assessment Confidence | Learning Effect | LE p-value | ICC-C |
| --- | --- | --- | --- | --- | --- | --- |
| Z_TaskScore | 0 | 1.42 | 1.01 | 0.27 | *0.02* | 0.64 |
| Z_DistractorHitsLeft | 0 | 1.85 | 1.31 | 0.15 | 0.29 | 0.43 |
| Z_DistractorHitsRight | 0 | 1.87 | 1.32 | 0.01 | 0.93 | 0.41 |
| Z_DistractorHitsTotal | 1 | 1.68 | 1.18 | 0.13 | 0.32 | 0.51 |
| Z_DistractorProportion | 0 | 1.75 | 1.24 | 0.11 | 0.43 | 0.52 |
| Z_HandBiasOfHits | 0 | 1.79 | 1.27 | 0.04 | 0.76 | 0.32 |
| Z_HandSelectionOverlap | 0 | 1.57 | 1.11 | 0.05 | 0.71 | 0.48 |
| Z_HandSpeedBias | 0 | 1.75 | 1.24 | 0.10 | 0.46 | 0.35 |
| Z_HandSpeedLeft | 0 | 1.10 | 0.78 | -0.19 | *0.03* | 0.74 |
| Z_HandSpeedRight | 0 | 1.20 | 0.85 | -0.12 | 0.22 | 0.72 |
| Z_HandTransition | 0 | 1.84 | 1.30 | 0.15 | 0.35 | 0.36 |
| Z_HitsWithLeft | 1 | 1.39 | 0.98 | 0.02 | 0.85 | 0.65 |
| Z_HitsWithRight | 0 | 1.51 | 1.07 | 0.09 | 0.47 | 0.52 |
| Z_M_score | 0 | 2.04 | 1.45 | 0.00 | 0.98 | 0.20 |
| Z_MedianError | 1 | 1.94 | 1.37 | -0.11 | 0.47 | 0.24 |
| Z_MissBias | 0 | 1.87 | 1.32 | 0.01 | 0.95 | 0.23 |
| Z_MovementAreaBias | 2 | 1.80 | 1.27 | -0.05 | 0.72 | 0.23 |
| Z_MovementAreaLeftHand | 0 | 1.36 | 0.96 | -0.03 | 0.79 | 0.57 |
| Z_MovementAreaRightHand | 0 | 1.24 | 0.88 | -0.05 | 0.62 | 0.71 |
| Z_ObjectProcessingRate | 0 | 1.35 | 0.95 | 0.01 | 0.91 | 0.62 |
| Z_ObjectsHit | 0 | 1.59 | 1.12 | 0.17 | 0.17 | 0.65 |
| Z_TotalHits | 0 | 1.21 | 0.86 | 0.08 | 0.41 | 0.75 |

LE (learning effect) p-values are italicized if <0.05 and have an asterisk if significant after false discovery rate (FDR) correction. ICC-C = intraclass correlation coefficient, consistency type.

Table ST4: PM-D

| Row | Outliers Removed | Significant Change | Assessment Confidence | Learning Effect | LE p-value | ICC-C | IS error |
| --- | --- | --- | --- | --- | --- | --- | --- |
| Z_TaskScore | 1 | 1.82 | 1.28 | -0.01 | 0.95 | 0.29 | NA |
| Z_AbsErrorX_m_ | 0 | 2.14 | 1.51 | 0.30 | 0.37 | 0.12 | ±0.09 |
| Z_AbsErrorXY_m_ | 3 | 1.78 | 1.26 | 0.06 | 0.73 | 0.23 | ±0.07 |
| Z_AbsErrorY_m_ | 2 | 1.81 | 1.28 | 0.31 | 0.22 | 0.27 | ±0.08 |
| Z_ContractionExpansionRatioX | 2 | 1.68 | 1.19 | 0.21 | 0.14 | 0.45 | NA |
| Z_ContractExpansionRatioXY | 0 | 1.42 | 1.00 | 0.16 | 0.19 | 0.58 | NA |
| Z_ContractionExpansionRatioY | 0 | 1.41 | 0.99 | -0.04 | 0.76 | 0.47 | NA |
| Z_M_score | 0 | 1.81 | 1.28 | -0.04 | 0.84 | 0.28 | NA |
| Z_ShiftX_m_ | 1 | 1.36 | 0.96 | 0.00 | 0.98 | 0.31 | NA |
| Z_ShiftXY_m_ | 0 | 1.77 | 1.25 | -0.01 | 0.93 | -0.01 | NA |
| Z_ShiftY_m_ | 1 | 1.33 | 0.94 | 0.06 | 0.57 | 0.31 | NA |
| Z_VariabilityX_m_ | 0 | 1.87 | 1.32 | -0.04 | 0.83 | 0.36 | NA |
| Z_VariabilityXY_m_ | 1 | 1.72 | 1.22 | -0.05 | 0.76 | 0.41 | NA |

LE (learning effect) p-values are italicized if <0.05 and have an asterisk if significant after false discovery rate (FDR) correction. ICC-C = intraclass correlation coefficient, consistency type. IS error = intra-subject error on the significant change derived from repeated trials. NA = Data not available.

Table ST5: PM-ND

| Row | Outliers Removed | Significant Change | Assessment Confidence | Learning Effect | LE p-value | ICC-C | IS error |
| --- | --- | --- | --- | --- | --- | --- | --- |
| Z_TaskScore | 0 | 1.72 | 1.21 | -0.01 | 0.94 | 0.36 | NA |
| Z_AbsErrorX_m_ | 0 | 2.45 | 1.73 | 0.25 | 0.19 | 0.21 | ±0.08 |
| Z_AbsErrorXY_m_ | 1 | 1.72 | 1.22 | 0.15 | 0.29 | 0.22 | ±0.08 |
| Z_AbsErrorY_m_ | 0 | 2.46 | 1.74 | -0.10 | 0.49 | 0.35 | ±0.09 |
| Z_ContractionExpansionRatioX | 1 | 1.51 | 1.07 | -0.13 | 0.24 | 0.49 | NA |
| Z_ContractExpansionRatioXY | 0 | 1.43 | 1.01 | -0.20 | 0.10 | 0.49 | NA |
| Z_ContractionExpansionRatioY | 0 | 1.95 | 1.38 | -0.20 | 0.13 | 0.58 | NA |
| Z_M_score | 0 | 1.83 | 1.29 | 0.05 | 0.67 | 0.40 | NA |
| Z_ShiftX_m | 0 | 1.76 | 1.24 | -0.07 | 0.54 | 0.46 | NA |
| Z_ShiftXY_m | 0 | 1.67 | 1.18 | 0.20 | 0.13 | 0.27 | NA |
| Z_ShiftY_m | 0 | 1.98 | 1.40 | 0.02 | 0.85 | 0.53 | NA |
| Z_VariabilityX_m | 1 | 1.91 | 1.35 | -0.10 | 0.51 | 0.35 | NA |
| Z_VariabilityXY_m | 0 | 1.83 | 1.29 | -0.12 | 0.45 | 0.41 | NA |

LE (learning effect) p-values are italicized if <0.05 and have an asterisk if significant after false discovery rate (FDR) correction. ICC-C = intraclass correlation coefficient, consistency type. IS error = intra-subject error on the significant change derived from repeated trials.

Table ST6: RVGR-D

| Row | Outliers Removed | Significant Change | Assessment Confidence | Learning Effect | LE p-value | ICC-C | IS error |
| --- | --- | --- | --- | --- | --- | --- | --- |
| Z_TaskScore | 0 | 1.34 | 0.95 | -0.78 | *<0.01** | 0.70 | NA |
| Z_CorrectionTime | 0 | 1.81 | 1.28 | -0.38 | 0.05 | 0.38 | NA |
| Z_InitialDirectionAngle | 3 | 1.84 | 1.30 | -0.74 | *<0.01** | 0.62 | ±0.15 |
| Z_InitialSpeedRatio | 1 | 1.63 | 1.15 | 0.70 | *<0.01** | 0.48 | ±0.14 |
| Z_M_score | 3 | 1.19 | 0.84 | -0.74 | *<0.01** | 0.77 | NA |
| Z_MaxSpeed | 0 | 1.09 | 0.77 | 0.59 | *<0.01** | 0.75 | NA |
| Z_MinMaxSpeedDifference | 1 | 1.20 | 0.85 | -0.08 | 0.43 | 0.73 | ±0.14 |
| Z_MovementTime | 0 | 1.02 | 0.72 | -0.76 | *<0.01** | 0.70 | ±0.07 |
| Z_PathLengthRatio | 2 | 1.72 | 1.21 | -0.41 | 0.05 | 0.76 | ±0.11 |
| Z_PostureSpeed | 2 | 1.42 | 1.00 | -0.31 | *0.02* | 0.56 | NA |
| Z_ReactionTime | 1 | 1.20 | 0.85 | -0.41 | *<0.01** | 0.87 | ±0.10 |
| Z_SpeedMaximaCount | 0 | 1.45 | 1.03 | -1.00 | *<0.01** | 0.65 | ±0.11 |

LE (learning effect) p-values are italicized if <0.05 and have an asterisk if significant after false discovery rate (FDR) correction. ICC-C = intraclass correlation coefficient, consistency type. IS error = intra-subject error on the significant change derived from repeated trials. NA = Data not available.

Table ST7: RVGR-ND

| Row | Outliers Removed | Significant Change | Assessment Confidence | Learning Effect | LE p-value | ICC-C | IS error |
| --- | --- | --- | --- | --- | --- | --- | --- |
| Z_TaskScore | 0 | 1.79 | 1.27 | -0.67 | *<0.01** | 0.67 | NA |
| Z_CorrectionTime | 0 | 2.31 | 1.64 | -0.59 | *<0.01** | 0.54 | NA |
| Z_InitialDirectionAngle | 2 | 2.81 | 1.99 | -0.73 | *<0.01** | 0.56 | ±0.16 |
| Z_InitialSpeedRatio | 4 | 2.20 | 1.55 | 0.63 | *<0.01** | 0.62 | ±0.15 |
| Z_M_score | 5 | 1.68 | 1.19 | -0.56 | *<0.01** | 0.74 | NA |
| Z_MaxSpeed | 0 | 1.51 | 1.07 | 0.25 | *0.01* | 0.91 | NA |
| Z_MinMaxSpeedDifference | 1 | 1.34 | 0.94 | -0.14 | 0.24 | 0.58 | ±0.15 |
| Z_MovementTime | 0 | 1.52 | 1.08 | -0.52 | *<0.01** | 0.70 | ±0.07 |
| Z_PathLengthRatio | 1 | 3.17 | 2.24 | -0.43 | 0.07 | 0.48 | ±0.12 |
| Z_PostureSpeed | 4 | 1.84 | 1.30 | -0.10 | 0.53 | 0.24 | NA |
| Z_ReactionTime | 0 | 1.59 | 1.12 | -0.36 | *<0.01** | 0.77 | ±0.10 |
| Z_SpeedMaximaCount | 0 | 2.40 | 1.70 | -0.69 | *<0.01** | 0.44 | ±0.11 |

LE (learning effect) p-values are italicized if <0.05 and have an asterisk if significant after false discovery rate (FDR) correction. ICC-C = intraclass correlation coefficient, consistency type. IS error = intra-subject error on the significant change derived from repeated trials. NA = Data not available.

Table ST8: SPS

| Row | Outliers Removed | Significant Change | Assessment Confidence | Learning Effect | LE p-value | ICC-C |
| --- | --- | --- | --- | --- | --- | --- |
| Z_TaskScore | 0 | 1.48 | 1.04 | -0.39 | *<0.01** | 0.56 |
| Z_M_score | 0 | 1.48 | 1.04 | -0.39 | *<0.01** | 0.56 |
| Z_MeanScore | 0 | 1.44 | 1.02 | 0.38 | *<0.01** | 0.56 |
| Z_TestTime | 0 | 1.60 | 1.13 | 0.44 | 0.20 | 0.30 |
| Z_TimePerTarget | 0 | 1.38 | 0.98 | -0.31 | *<0.01** | 0.66 |
| Z_TotalScore | 1 | 1.44 | 1.02 | 0.38 | *<0.01** | 0.56 |

LE (learning effect) p-values are italicized if <0.05 and have an asterisk if significant after false discovery rate (FDR) correction. ICC-C = intraclass correlation coefficient, consistency type.

.

Table ST9: TMT

| Row | Outliers Removed | Significant Change | Assessment Confidence | Learning Effect | LE p-value | ICC-C |
| --- | --- | --- | --- | --- | --- | --- |
| Z_TaskScore | 0 | 0.87 | 0.61 | -0.23 | *<0.01** | 0.75 |
| Z_B_A | 0 | 1.08 | 0.76 | -0.34 | *<0.01** | 0.60 |
| Z_DwellTime | 1 | 0.85 | 0.60 | -0.34 | *<0.01** | 0.75 |
| Z_M_score | 0 | 1.18 | 0.83 | 0.07 | 0.46 | 0.62 |
| Z_TestTime | 0 | 0.92 | 0.65 | -0.28 | *<0.01** | 0.69 |
| Z_TimeRatio | 0 | 1.94 | 1.37 | -0.26 | 0.11 | 0.07 |

LE (learning effect) p-values are italicized if <0.05 and have an asterisk if significant after false discovery rate (FDR) correction. ICC-C = intraclass correlation coefficient, consistency type.

Table ST10: VGR-D

| Row | Outliers Removed | Significant Change | Assessment Confidence | Learning Effect | LE p-value | ICC-C | IS error |
| --- | --- | --- | --- | --- | --- | --- | --- |
| Z_TaskScore | 0 | 1.84 | 1.30 | -0.23 | 0.17 | 0.34 | NA |
| Z_InitialDirectionAngle | 0 | 1.90 | 1.34 | -0.24 | 0.14 | 0.25 | ±0.17 |
| Z_InitialDistanceRatio | 0 | 1.44 | 1.01 | 0.35 | *0.03* | 0.20 | NA |
| Z_M_score | 1 | 1.61 | 1.14 | -0.15 | 0.30 | 0.54 | NA |
| Z_MaxSpeed | 1 | 1.02 | 0.72 | 0.04 | 0.65 | 0.85 | NA |
| Z_MinMaxSpeedDifference | 0 | 1.42 | 1.00 | -0.28 | *0.04* | 0.54 | ±0.11 |
| Z_MovementTime | 1 | 1.44 | 1.02 | -0.16 | 0.19 | 0.64 | ±0.19 |
| Z_PathLengthRatio | 0 | 1.64 | 1.16 | -0.34 | *0.03* | 0.57 | ±0.06 |
| Z_PostureSpeed | 1 | 1.79 | 1.27 | -0.12 | 0.48 | 0.35 | NA |
| Z_ReactionTime | 1 | 1.34 | 0.95 | 0.02 | 0.85 | 0.73 | ±0.21 |
| Z_SpeedMaximaCount | 0 | 1.74 | 1.23 | -0.43 | *0.01* | 0.14 | ±0.23 |

LE (learning effect) p-values are italicized if <0.05 and have an asterisk if significant after false discovery rate (FDR) correction. ICC-C = intraclass correlation coefficient, consistency type. IS error = intra-subject error on the significant change derived from repeated trials. NA = Data not available.

Table ST11: VGR-ND

| Row | Outliers Removed | Significant Change | Assessment Confidence | Learning Effect | LE p-value | ICC-C | IS error |
| --- | --- | --- | --- | --- | --- | --- | --- |
| Z_TaskScore | 0 | 2.19 | 1.55 | -0.17 | 0.31 | 0.33 | NA |
| Z_InitialDirectionAngle | 0 | 2.08 | 1.47 | -0.45 | *<0.01** | 0.26 | ±0.17 |
| Z_InitialDistanceRatio | 2 | 2.00 | 1.41 | 0.33 | 0.08 | -0.05 | NA |
| Z_M_score | 1 | 1.93 | 1.37 | -0.07 | 0.64 | 0.53 | NA |
| Z_MaxSpeed | 1 | 1.07 | 0.76 | -0.03 | 0.71 | 0.84 | NA |
| Z_MinMaxSpeedDifference | 0 | 1.66 | 1.17 | -0.27 | *0.05* | 0.60 | ±0.11 |
| Z_MovementTime | 2 | 1.52 | 1.07 | -0.04 | 0.68 | 0.61 | ±0.19 |
| Z_PathLengthRatio | 0 | 1.73 | 1.22 | -0.37 | *<0.01** | 0.70 | ±0.06 |
| Z_PostureSpeed | 1 | 1.99 | 1.41 | -0.07 | 0.65 | 0.40 | NA |
| Z_ReactionTime | 1 | 1.23 | 0.87 | -0.11 | 0.34 | 0.70 | ±0.23 |
| Z_SpeedMaximaCount | 0 | 2.06 | 1.46 | -0.23 | 0.13 | 0.34 | ±0.22 |

LE (learning effect) p-values are italicized if <0.05 and have an asterisk if significant after false discovery rate (FDR) correction. ICC-C = intraclass correlation coefficient, consistency type. IS error = intra-subject error on the significant change derived from repeated trials. NA = Data not available.
